# Supplementary material for: A novel signature derived from immunoregulatory and hypoxia genes predicts prognosis in liver and five other cancers
Source: J Transl Med. 2019 Jan 9;17:14. doi: 10.1186/s12967-019-1775-9 (PMC6327401; doi:10.1186/s12967-019-1775-9)
Supplement: Supplementary file 1 — Additional file 1. Abbreviations and number of tumor and non-tumor samples in TCGA cancers. [file 12967_2019_1775_MOESM1_ESM.pdf]

Additional file 1. Abbreviations and number of tumour and non-tumour samples in TCGA cancers.

Cohort (TCGA abbreviations)	Non-tumour #	Tumour #	Description
BLCA	19	408	Bladder Urothelial Carcinoma
BRCA	112	10939	Breast invasive carcinoma
CESC	3	304	Cervical squamous cell carcinoma and endocervical adenocarcinoma
CHOL	9	36	Cholangiocarcinoma
COAD	41	285	Colon adenocarcinoma
ESCA	11	184	Esophageal carcinoma
GBM	5	153	Glioblastoma multiforme
GBMLGG	5	669	Glioma
HNSC	44	520	Head and Neck squamous cell carcinoma
KICH	25	66	Kidney Chromophobe
KIPAN	129	889	Pan-kidney cohort
KIRC	72	533	Kidney renal clear cell carcinoma
KIRP	32	290	Kidney renal papillary cell carcinoma
LIHC	50	371	Liver hepatocellular carcinoma
LUAD	59	515	Lung adenocarcinoma
LUSC	51	501	Lung squamous cell carcinoma
PAAD	4	178	Pancreatic adenocarcinoma
PCPG	3	179	Pheochromocytoma and Paraganglioma
PRAD	52	497	Prostate adenocarcinoma
SARC	2	259	Sarcoma
STAD	35	415	Stomach adenocarcinoma
STES	46	599	Stomach and Esophageal carcinoma
THCA	59	501	Thyroid carcinoma
THYM	2	120	Thymoma
UCEC	11	370	Uterine Corpus Endometrial Carcinoma
