# Supplementary material for: A novel signature derived from immunoregulatory and hypoxia genes predicts prognosis in liver and five other cancers
Source: J Transl Med. 2019 Jan 9;17:14. doi: 10.1186/s12967-019-1775-9 (PMC6327401; doi:10.1186/s12967-019-1775-9)
Supplement: Supplementary file 2 — Additional file 2. Clinical characteristics of HCC patients. [file 12967_2019_1775_MOESM2_ESM.pdf]

Additional file 2. Clinical characteristics of HCC patients (n = 839)

Characteristic		GSE14520	TCGA-LIHC	LIRI-JP
Patient number	Total	242	371	226
	Male	211 (87%)	252 (68%)	166 (73%)
	Female	31 (13%)	119 (32%)	60 (27%)
Age (years)	Range	21 - 77	16 - 90	31 - 89
	Median	50	61	68
Viral etiology	Hepatitis B	242 (100%)	143 (39%)	59 (26%)
	Hepatitis C		44 (12%)	120 (53%)
	Hepatitis B & C		87 (24%)	4 (2%)
	Negative		7 (2%)	43 (19%)
	Not available		90 (23%)	
Edmondson grade	I			28 (12%)
	II			156 (69%)
	III			21 (9%)
	IV			1 (0.4%)
	Not available	242 (100%)	371 (100%)	20 (9%)
Tumour size	Small (< 5cm)	153 (63%)		174 (77%)
	Large (> 5cm)	89 (37%)		52 (23%)
	Not available		371 (100%)	
Fibrosis	Negative	19 (8%)	76 (20%)	21 (9%)
	Portal fibrosis (stages 1 -2)		31 (8%)	73 (32%)
	Fibrous septa (stages 3 - 4)		30 (8%)	132 (58%)
	Nodular formation (stage 5)		9 (2%)	
	Cirrhosis (stage 6)	223 (92%)	72 (19%)	
	Not available		153 (41%)	
Alcohol consumption	Yes		118 (32%)	86 (38%)
	No			128 (57%)
	Not available	242 (100%)	253 (68%)	12 (5%)
Smoker	Yes		13 (4%)	122 (54%)
	No			94 (42%)
	Not available	242 (100%)	358 (96%)	10 (4%)
AFP	Low (< 300ng/mL)	128 (53%)	218 (59%)	
	High (> 300ng/mL)	110 (45%)	66 (18%)	
	Not available	4 (2%)	87 (23%)	226 (100%)
BCLC stage	0	20 (8%)		
	A	152 (63%)		
	B	24 (10%)		
	C	29 (12%)		
	Not available	17 (7%)	371 (100%)	226 (100%)
TNM stage	I	96 (40%)	144 (39%)	35 (15%)
	II	78 (32%)	67 (18%)	101 (45%)
	III	51 (21%)	71 (19%)	68 (30%)
	IV			18 (8%)
	Not available	17 (7%)	89 (24%)	4 (2%)
