# Supplementary figures and images for: A novel signature derived from immunoregulatory and hypoxia genes predicts prognosis in liver and five other cancers
Source: J Transl Med. 2019 Jan 9;17:14. doi: 10.1186/s12967-019-1775-9 (PMC6327401; doi:10.1186/s12967-019-1775-9)

Additional file 3

A

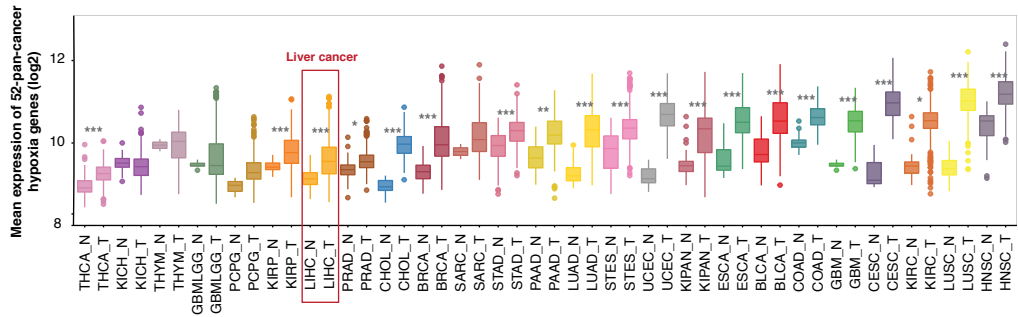

B

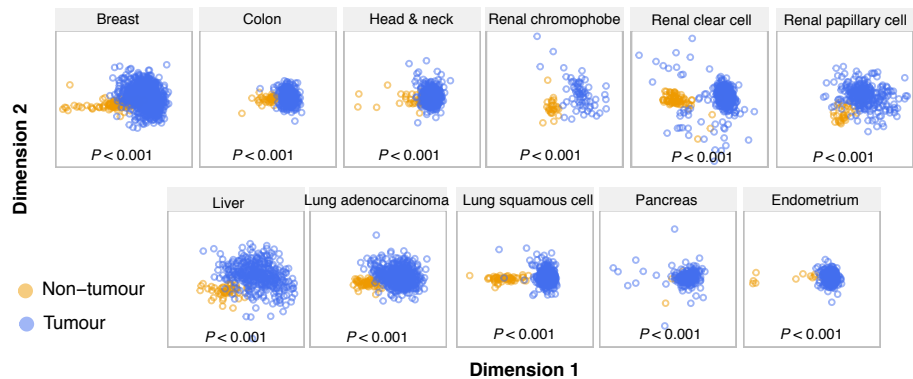

C

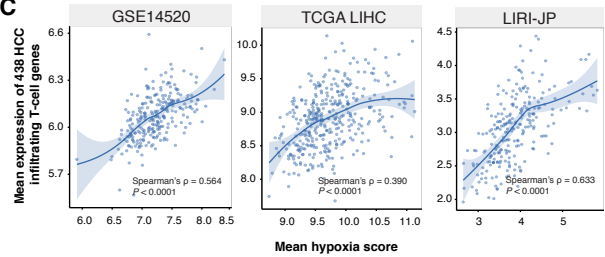

D

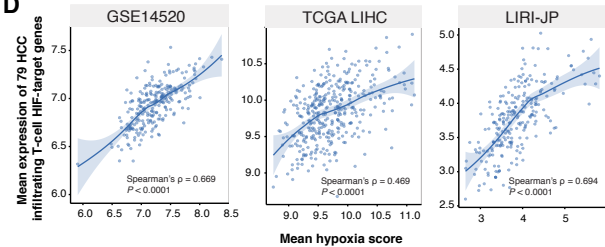

E

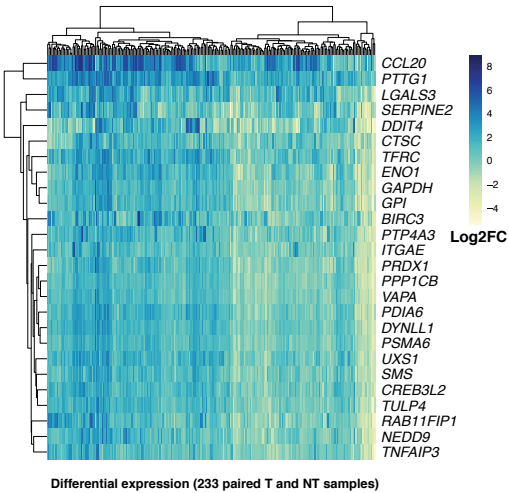

Supplement: Supplementary file 3 — Additional file 3. Genes associated with tumor hypoxia and T-cell infiltration. (A) Box plot depicts hypoxia score between tumor (T) and non-tumor (N) samples across 25 cancer datasets obtained from TCGA. Hypoxia scores were estimated by obtaining the mean expression (log2) of 52 hypoxia genes reported by Buffa et al. (2010). Cancer types were sorted on the basis of smallest to largest median hypoxia score in tumor samples. Distribution of hypoxia scores for T and NT samples for each cancer was compared using the Mann-Whitney-Wilcoxon test. Asterisks represent significant P values: * < 0.01, ** < 0.001 and *** < 0.0001. TCGA abbreviations were used to represent cancer types; refer to Additional file 1. (B) Ordination plots of multidimensional scaling analysis of the 52 hypoxia signature genes using Euclidean distances revealed significant separation of tumor (T) and non-tumor (NT) samples represented in a 2-dimensional space. Axes represent the first and second dimension. The distinction of T and NT was confirmed by permutational multivariate analysis of variance (PERMANOVA) tests. Analysis was performed using the metaMDS and adonis function of the R vegan package. (C and D) Significant positive correlation between HCC-infiltrating gene expression and tumor hypoxia. Expression of both (C) 438 gene set (HCC infiltrating T cells) and (D) 79 gene set (HIF-target genes associated with HCC infiltrating T-cells) positively correlated with tumor hypoxia as determined from the Buffa hypoxia gene signature. (E) Heatmap depicts differential expression values of 26 HCC-upregulated genes in 233 tumor and non-tumor paired samples from the training cohort. Of the 79 HIF-target genes associated with HCC infiltrating T-cells, 26 are at least 1.5-fold significantly upregulated. [file 12967_2019_1775_MOESM3_ESM.pdf]

Additional file 5

A

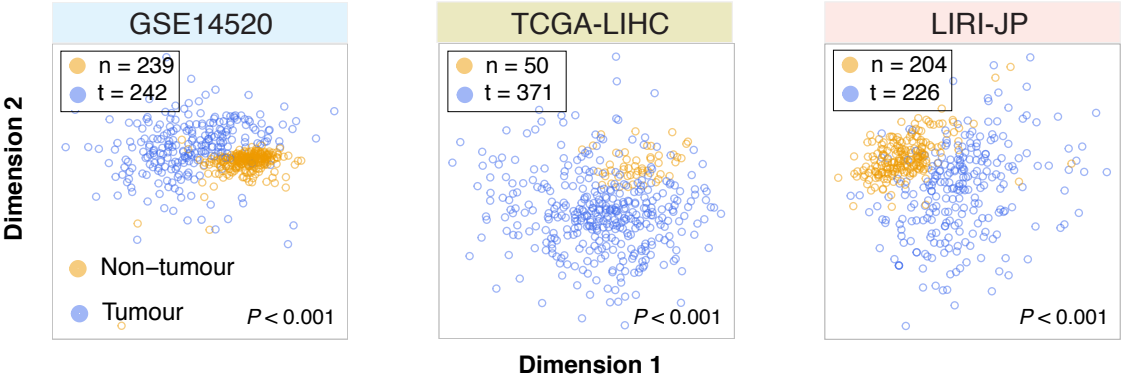

B

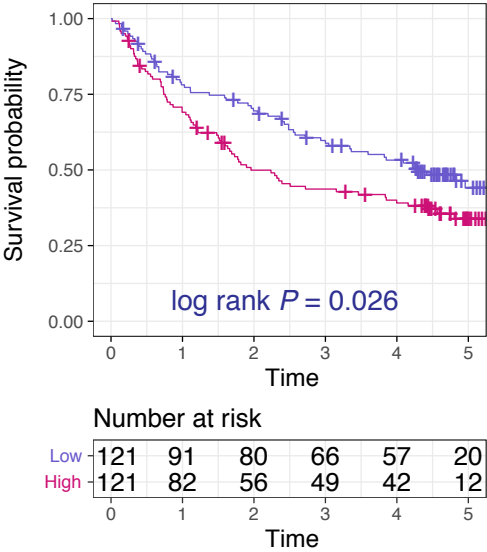

Supplement: Supplementary file 5 — Additional file 5. Multidimensional scaling analysis of the 45-gene signature and disease-free survival analysis. (A) Ordination plots of multidimensional scaling analysis of the signature in HCC cohorts using Euclidean distances revealed significant separation of tumor (T) and non-tumor (NT) samples represented in a 2-dimensional space. Axes represent the first and second dimension. The distinction of T and NT was confirmed by PERMANOVA tests. (B) Kaplan-Meier plot of disease-free survival in HCC patients from the GSE14520 cohort stratified into low- and high-risk groups using the 45-gene signature. Disease-free survival is defined as the time from surgery to recurrence, death from any cause or distant metastasis. P-values are calculated from the log-rank test. [file 12967_2019_1775_MOESM5_ESM.pdf]

A

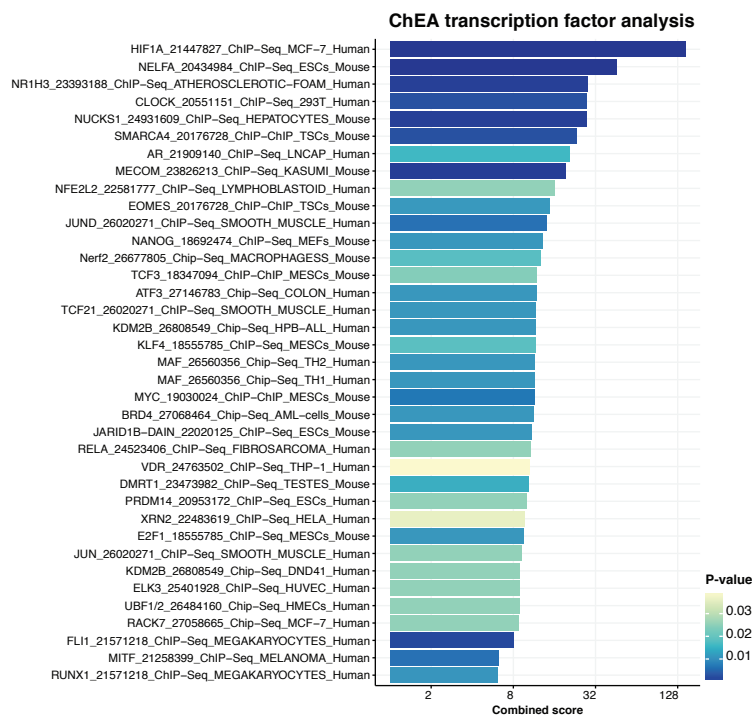

B

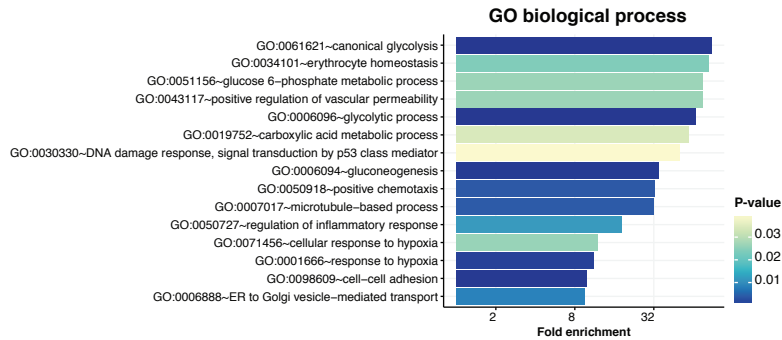

C

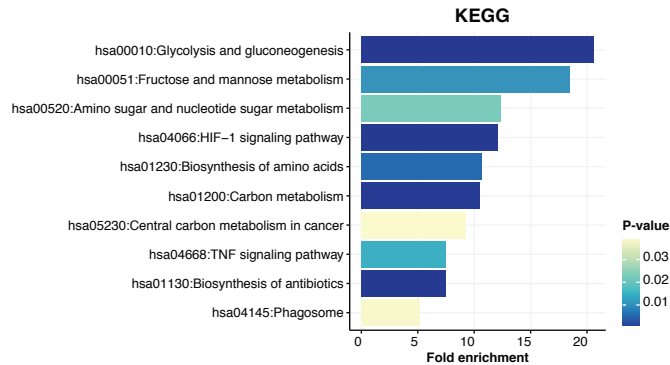

Supplement: Supplementary file 6 — Additional file 6. Biological functions associated with the 45-gene signature revealed enrichments of pathways associated with hypoxia, metabolism and cancer. (A) Transcription factors and histone modifiers that are potential regulators of the 45 genes. (B) Enrichment of GO biological processes. (C) Enrichment of KEGG ontologies. [file 12967_2019_1775_MOESM6_ESM.pdf]

### Additional file 7

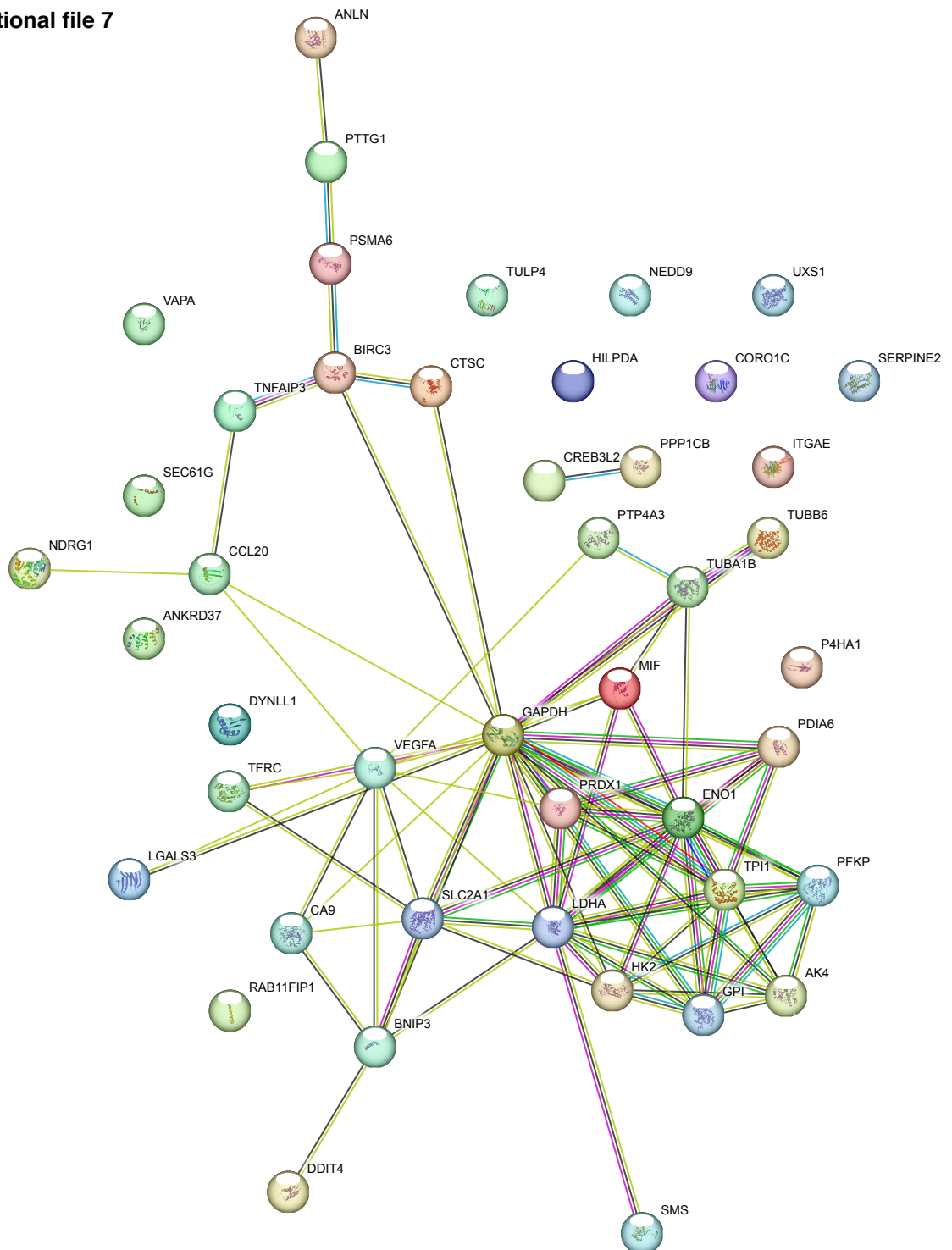

Protein-protein interaction enrichment, P-value &lt; 1.0e-16

Supplement: Supplementary file 7 — Additional file 7. Protein-protein interaction (PPI) networks associated with the 45-gene signature. As determined by STRING (version 10.5), PPI enrichment was significant (P < 1e-16) indicating that the proteins are biologically connected as a group. [file 12967_2019_1775_MOESM7_ESM.pdf]

## Additional file 9

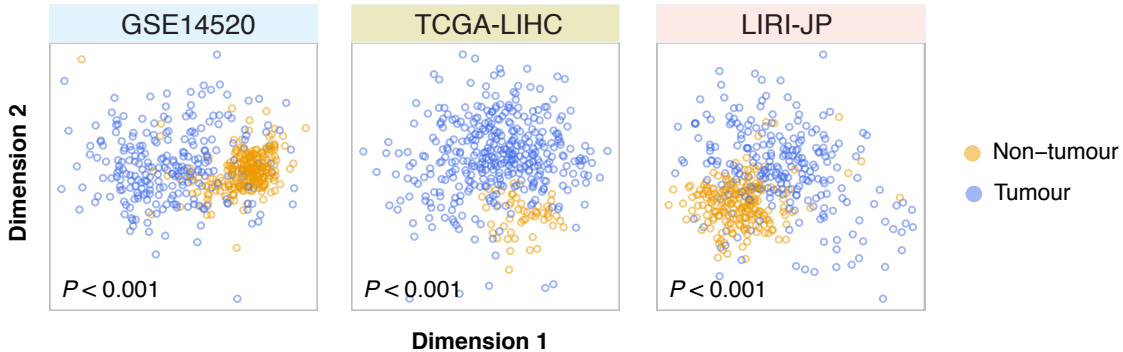

Supplement: Supplementary file 9 — Additional file 9. Ordination plots of multidimensional scaling analysis of the 8-gene signature in HCC cohorts using Euclidean distances revealed significant separation of tumor (T) and non-tumor (NT) samples represented in a 2-dimensional space. Axes represent the first and second dimension. The distinction of T and NT was confirmed by PERMANOVA tests. [file 12967_2019_1775_MOESM9_ESM.pdf]

# Additional file 10

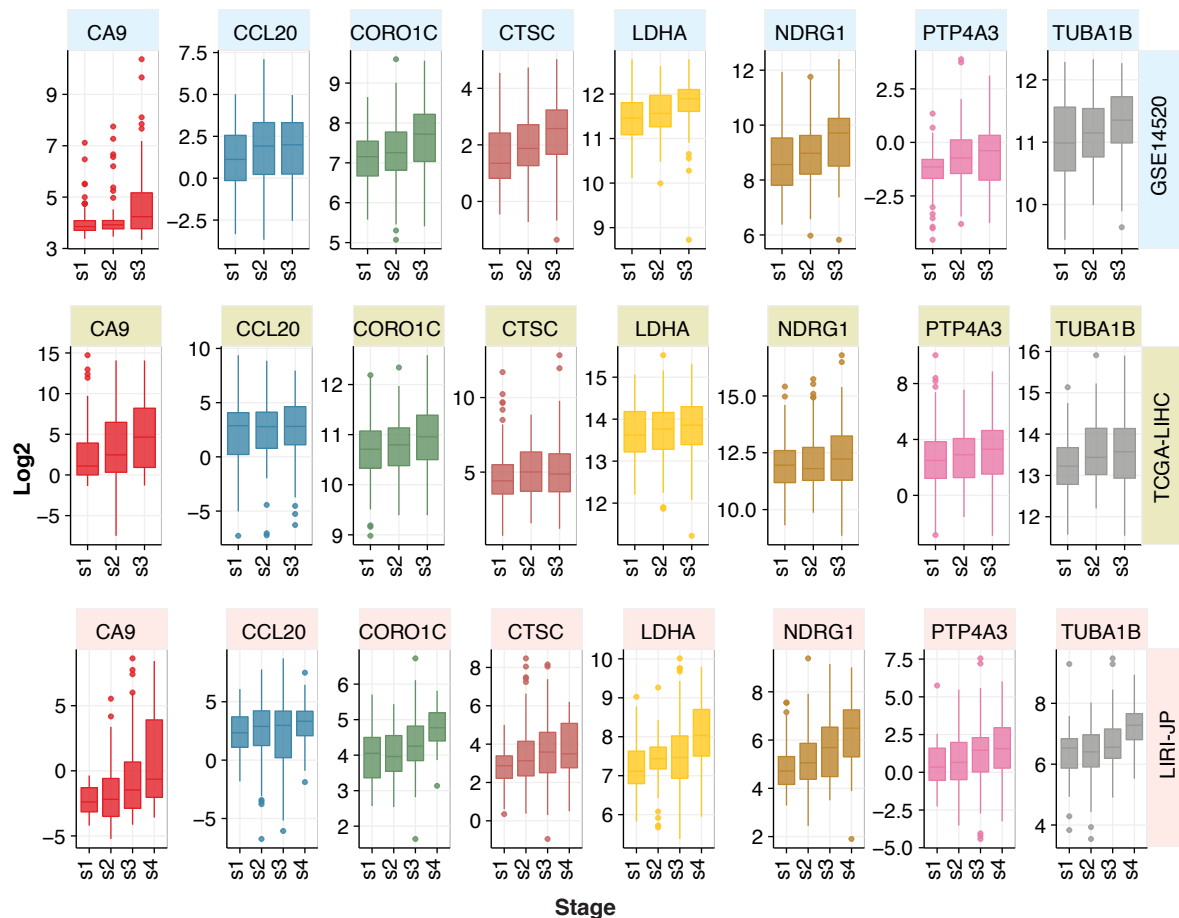

Supplement: Supplementary file 10 — Additional file 10. Expression distribution of genes from the 8-gene signature according to tumor staging in three HCC cohorts. Expression of genes increased with tumor staging in HCC patients. [file 12967_2019_1775_MOESM10_ESM.pdf]

Additional file 11

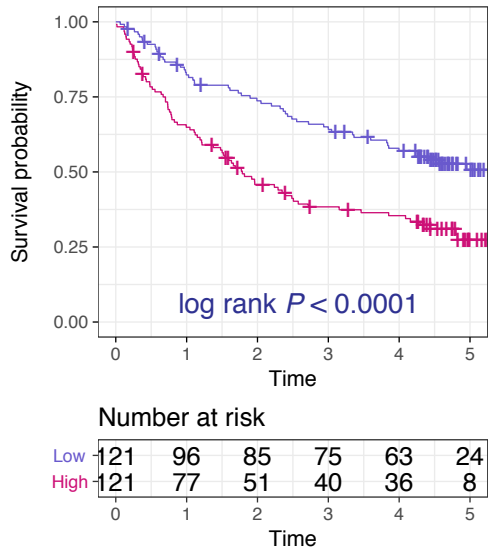

Supplement: Supplementary file 11 — Additional file 11. Kaplan-Meier plot of disease-free survival in HCC patients from the GSE14520 cohort stratified into low- and high-risk groups using the 8-gene signature. Disease-free survival is defined as the time from surgery to recurrence, death from any cause or distant metastasis. P-values are calculated from the log-rank test. [file 12967_2019_1775_MOESM11_ESM.pdf]

## Additional file 12

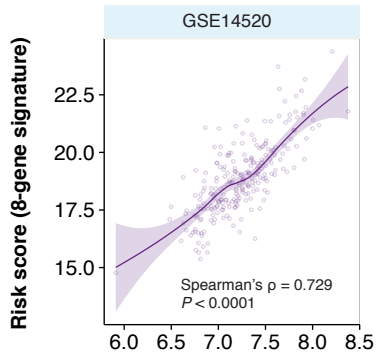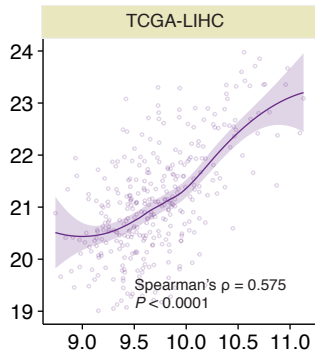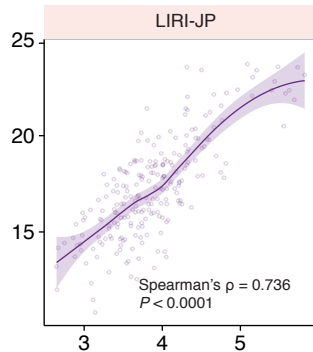

**Mean hypoxia score**

Supplement: Supplementary file 12 — Additional file 12. Correlation of risk scores, as determined using the 8-gene signature, and hypoxia scores in HCC patients. Significant positive correlation between patient survival risk scores (refer to “Methods”) derived from the 8-gene signature and tumor hypoxia in HCC cohorts. [file 12967_2019_1775_MOESM12_ESM.pdf]

## Additional file 13

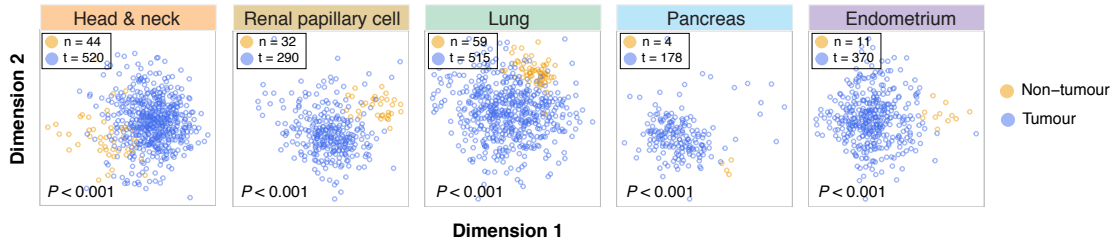

Supplement: Supplementary file 13 — Additional file 13. Ordination plots of multidimensional scaling analysis of the 8-gene signature in cancers using Euclidean distances revealed significant separation of tumor (T) and non-tumor (NT) samples represented in a 2-dimensional space. Axes represent the first and second dimension. The distinction of T and NT was confirmed by PERMANOVA tests. [file 12967_2019_1775_MOESM13_ESM.pdf]

## Additional file 15

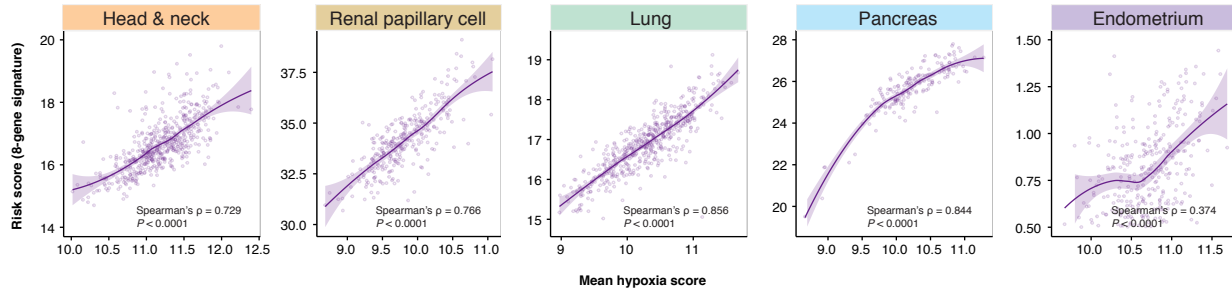

Supplement: Supplementary file 15 — Additional file 15. Correlation of risk scores, as determined using the 8-gene signature, and hypoxia scores in other cancers. Significant positive correlation between patient survival risk scores (refer to “Methods”) derived from the 8-gene signature and tumor hypoxia in cancers. [file 12967_2019_1775_MOESM15_ESM.pdf]
