# Supplementary material for: A novel signature derived from immunoregulatory and hypoxia genes predicts prognosis in liver and five other cancers
Source: J Transl Med. 2019 Jan 9;17:14. doi: 10.1186/s12967-019-1775-9 (PMC6327401; doi:10.1186/s12967-019-1775-9)
Supplement: Supplementary file 4 — Additional file 4. The 45-gene signature associated with tumor hypoxia and T-cell infiltration. [file 12967_2019_1775_MOESM4_ESM.pdf]

Additional file 4. The 45-gene classifier associated with tumour hypoxia and T-cell infiltration.

Gene Symbol	Description
AK4	adenylate kinase 4
ANKRD37	ankyrin repeat domain 37
ANLN	anillin actin binding protein
BIRC3	baculoviral IAP repeat containing 3
BNIP3	BCL2 interacting protein 3
CA9	carbonic anhydrase 9
CCL20	C-C motif chemokine ligand 20
CORO1C	coronin 1C
CREB3L2	cAMP responsive element binding protein 3 like 2
CTSC	cathepsin C
DDIT4	DNA damage inducible transcript 4
DYNLL1	dynein light chain LC8-type 1
ENO1	enolase 1
GAPDH	glyceraldehyde-3-phosphate dehydrogenase
GPI	glucose-6-phosphate isomerase
HILPDA	hypoxia inducible lipid droplet associated
HK2	hexokinase 2
ITGAE	integrin subunit alpha E
LDHA	lactate dehydrogenase A
LGALS3	galectin 3
MIF	macrophage migration inhibitory factor
NDRG1	N-myc downstream regulated 1
NEDD9	neural precursor cell expressed, developmentally down-regulated 9
P4HA1	prolyl 4-hydroxylase subunit alpha 1
PDIA6	protein disulfide isomerase family A member 6
PFKP	phosphofructokinase, platelet
PPP1CB	protein phosphatase 1 catalytic subunit beta
PRDX1	peroxiredoxin 1
PSMA6	proteasome subunit alpha 6
PTP4A3	protein tyrosine phosphatase type IVA, member 3
PTTG1	pituitary tumor-transforming 1
RAB11FIP1	RAB11 family interacting protein 1
SEC61G	Sec61 translocon gamma subunit
SERPINE2	serpin family E member 2
SLC2A1	solute carrier family 2 member 1
SMS	spermine synthase
TFRC	transferrin receptor
TNFAIP3	TNF alpha induced protein 3
TPI1	triosephosphate isomerase 1
TUBA1B	tubulin alpha 1b
TUBB6	tubulin beta 6 class V
TULP4	tubby like protein 4
UXS1	UDP-glucuronate decarboxylase 1
VAPA	VAMP associated protein A
VEGFA	vascular endothelial growth factor A
