# Supplementary material for: A novel signature derived from immunoregulatory and hypoxia genes predicts prognosis in liver and five other cancers
Source: J Transl Med. 2019 Jan 9;17:14. doi: 10.1186/s12967-019-1775-9 (PMC6327401; doi:10.1186/s12967-019-1775-9)
Supplement: Supplementary file 8 — Additional file 8. The minimal prognostic 8-gene signature. [file 12967_2019_1775_MOESM8_ESM.pdf]

**Additional file 8. The minimal prognostic 8-gene classifier.**

| Gene Symbol | Description |
|---|---|
| CA9 | carbonic anhydrase 9 |
| CCL20 | C-C motif chemokine ligand 20 |
| CORO1C | coronin 1C |
| CTSC | cathepsin C |
| LDHA | lactate dehydrogenase A |
| NDRG1 | N-myc downstream regulated 1 |
| PTP4A3 | protein tyrosine phosphatase type IVA, member 3 |
| TUBA1B | tubulin alpha 1b |
