# Supplementary material for: A novel signature derived from immunoregulatory and hypoxia genes predicts prognosis in liver and five other cancers
Source: J Transl Med. 2019 Jan 9;17:14. doi: 10.1186/s12967-019-1775-9 (PMC6327401; doi:10.1186/s12967-019-1775-9)
Supplement: Supplementary file 14 — Additional file 14. Univariate and multivariate Cox proportional hazards analysis of risk factors associated with overall survival in five non-HCC cancers. Significant covariates are highlighted in bold. [file 12967_2019_1775_MOESM14_ESM.pdf]

**Additional file 14. Univariate and multivariate Cox proportional hazards analysis of risk factors associated with overall survival in multiple cancers.**

| | Univariate | | Multivariate | |
|---|---|---|---|---|
| | **Hazard Ratio (95% CI)** | ***P*-value** | **Hazard Ratio (95% CI)** | ***P*-value** |
| **Head and neck** | | | | |
| 8-gene signature (high vs. low risk) | 1.64 (1.166 - 2.308) | **4.70E-03** | 1.658 (1.178 - 2.322) | **3.74E-03** |
| TNM staging (II & III vs. I) | 1.466 (1.188 - 1.808) | **3.53E-04** | 1.475 (1.194 - 1.820) | **3.05E-04** |
| *TP63* mutation (Yes vs. No) | 1.046 (0.658 - 1.663) | 0.85 | | |
| *CDKN2A* mutation (Yes vs. No) | 1.318 (0.892 - 1.947) | 0.17 | | |
| *PIK3CA* mutation (Yes vs. No) | 1.261 (0.785 - 2.026) | 0.34 | | |
| **Renal papillary cell** | | | | |
| 8-gene signature (high vs. low risk) | 2.311 (1.097 - 5.484) | **0.0475** | 1.190 (0.4771 - 2.970) | 0.709 |
| TNM staging (II & III vs. I) | 2.71 (1.893 - 3.878) | **5.30E-08** | 2.647 (1.815 - 3.861) | **4.35E-07** |
| **Lung** | | | | |
| 8-gene signature (high vs. low risk) | 1.453 (1.036 - 2.038) | **0.0304** | 1.458 (1.041 - 2.043) | **0.0281** |
| TNM staging (II & III vs. I) | 1.597 (1.364 - 1.87) | **6.11E-09** | 1.598 (1.362 - 1.873) | **8.04E-09** |
| *TP53* mutation (Yes vs. No) | 1.316 (0.863 - 2.006) | 0.203 | | |
| *KRAS* mutation (Yes vs. No) | 0.764 (0.401 - 1.455) | 0.412 | | |
| *CDKN2A* mutation (Yes vs. No) | 1.759 (1.057 - 2.927) | **0.030** | 1.533 (0.920 - 2.554) | 0.101 |
| **Pancreas** | | | | |
| 8-gene signature (high vs. low risk) | 1.961 (1.212 - 3.174) | **0.00609** | 1.906 (1.177 - 3.089) | **0.00878** |
| TNM staging (II & III vs. I) | 1.339 (0.897 - 1.998) | 0.153 | 1.282 (0.835 - 1.967) | 0.255 |
| **Endometrium** | | | | |
| 8-gene signature (high vs. low risk) | 2.33 (1.341 - 4.05) | **0.0027** | 1.986 (1.136 - 3.472) | **0.016** |
| TNM staging (II & III vs. I) | 1.802 (1.431 - 2.269) | **5.60E-07** | 1.722 (1.365 - 2.172) | **4.56E-06** |
