# Supplementary material for: A novel signature derived from immunoregulatory and hypoxia genes predicts prognosis in liver and five other cancers
Source: J Transl Med. 2019 Jan 9;17:14. doi: 10.1186/s12967-019-1775-9 (PMC6327401; doi:10.1186/s12967-019-1775-9)
Supplement: Supplementary file 16 — Additional file 16. Kaplan-Meier plot depicting combined relation of CDKN2A mutation status with the 8-gene signature on overall survival in lung cancer. [file 12967_2019_1775_MOESM16_ESM.pdf]

Additional file 16

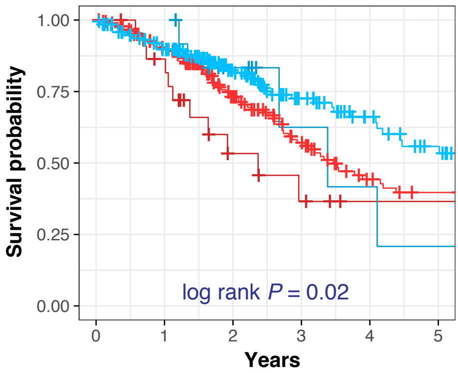

Number at risk

High Risk, *CDKN2A* mutant	23	18	7	4	1	1
High Risk, *CDKN2A* WT	185	152	84	52	30	24
Low Risk, *CDKN2A* mutant	13	13	7	3	2	1
Low Risk, *CDKN2A* WT	194	158	89	52	34	23
